# Temporal Estimation of Non-Rigid Dynamic Human Point Cloud Sequence Using 3D Skeleton-Based Deformation for Compression

**DOI:** 10.3390/s23167163

**Published:** 2023-08-14

**Authors:** Jin-Kyum Kim, Ye-Won Jang, Sol Lee, Eui-Seok Hwang, Young-Ho Seo

**Affiliations:** 1 Electronic Materials Engineering, Kwangwoon University, Kwangwoon-ro 20, Seoul 01897, Republic of Korea; jkkim@kw.ac.kr (J.-K.K.); ywjang@kw.ac.kr (Y.-W.J.); solee@kw.ac.kr (S.L.); 2 Yeshcompany, 4, Nonhyeon-ro 64-gil, Gangnam-gu, Seoul 06231, Republic of Korea; ushwang@yesh.co.kr

**Keywords:** dynamic point cloud, augmented reality, virtual reality, pose estimation, 3D skeleton, deformation, temporal prediction

## Abstract

This paper proposes an algorithm for transmitting and reconstructing the estimated point cloud by temporally estimating a dynamic point cloud sequence. When a non-rigid 3D point cloud sequence (PCS) is input, the sequence is divided into groups of point cloud frames (PCFs), and a key PCF is selected. The 3D skeleton is predicted through 3D pose estimation, and the motion of the skeleton is estimated by analyzing the joints and bones of the 3D skeleton. For the deformation of the non-rigid human PC, the 3D PC model is transformed into a mesh model, and the key PCF is rigged using the 3D skeleton. After deforming the key PCF into the target PCF utilizing the motion vector of the estimated skeleton, the residual PC between the motion compensation PCF and the target PCF is generated. If there is a key PCF, the motion vector of the target PCF, and a residual PC, the target PCF can be reconstructed. Just as compression is performed using pixel correlation between frames in a 2D video, this paper compresses 3D PCFs by estimating the non-rigid 3D motion of a 3D object in a 3D PC. The proposed algorithm can be regarded as an extension of the 2D motion estimation of a rigid local region in a 2D plane to the 3D motion estimation of a non-rigid object (human) in 3D space. Experimental results show that the proposed method can successfully compress 3D PC sequences. If it is used together with a PC compression technique such as MPEG PCC (point cloud compression) in the future, a system with high compression efficiency may be configured.

## 1. Introduction

With the recent development of virtual reality (VR) and augmented reality (AR) technologies, 3D volumetric content technology that provides a realistic experience from a free point of view has been actively developed [1]. Three-dimensional volumetric content can be applied to various applications such as games, video services, medical care, and education. When a 3D volumetric model is consumed through a device, like other contents, coding-related technology for efficient transmission and data storage is emerging as an important issue [2]. A point cloud (PC), which expresses the surface of an object in the form of a point, is typically used to express volumetric 3D data [1]. These data contain 3D coordinates and texture color for each point, and additional information such as shape, normal vectors, and maps is included depending on the application field. These data require a more massive amount of bits than a 2D image because it uses hundreds of thousands or millions of points for visualization. Therefore, coding is essential for 3D PC [3].

We introduce some previous research to compress 3D PC [3]. The first is a method to compress 2D images projected from a 3D PC. First, the virtual cubic box, including an object, is defined in 3D space to map an object into a virtual cubic box. Then, the points of an object are projected into corresponding planes using their normal vectors. This process is regarded as making patches by clustering 3D PC. Finally, each patch is cast into the nearest virtual plane and placed in a rectangular atlas [4,5]. Two images with geometry and texture information are output for each frame, and a video codec compresses the resultant sequence.

There was a method to compress the point cloud using the octree. The sparse voxel octree was first used to express the geometry of a 3D object [6,7], but it was also used to compress the point cloud using octree serialization [8]. In the intra-coding, the data can be reduced by removing the spatially overlapped point clouds [9]. In the inter-coding, the temporally repeated point clouds are removed using the XOR arithmetic of the octree serialization between frames [10,11].

The motion estimation and compensation algorithm for enhancing the coding efficiency of a video sequence can be applied to the compression of the point cloud. The motion estimation of the point cloud divides the point cloud into voxel blocks and finds the motion vector of each voxel block [12]. Another method draws vertex graphs of 3D coordinates and the texture of the point cloud and estimates the motion vector of vertices using spectral graph wavelet descriptors [13].

We propose a method of compressing the point cloud sequence by extracting the 3D skeleton of the point cloud. The skeleton is generated based on OpenPose, a deep learning model for 2D pose estimation. Since the quality of the extracted 3D skeleton significantly affects the point cloud compression performance, a 3D skeleton with high precision is required. Point cloud compression is performed by deforming the key frame point cloud using the motion vector of each joint of the skeleton of the non-key frame and removing overlapping points between the deformed key frame and the point cloud of the target frame. For point cloud deformation, key frame point clouds are converted to mesh models for rigging. The interval at which the key frame is determined depends on the target compression rate.

This paper is structured as follows. Section 2 explains the process of acquiring a point cloud sequence based on reality. Section 3 describes the 3D pose estimation technique, and a point cloud sequence compression algorithm is proposed. Section 4 shows the compression result by the proposed algorithm, and Section 5 concludes this paper.

## 2. Dynamic Point Cloud Sequence

In this section, we explain the 3D point cloud sequence. First, we describe the point cloud structure and the method to capture the 3D point cloud. Next, we introduce a way to precisely estimate 3D human pose estimation using a 3D point cloud.

### 2.1. Dynamic Point Cloud

To generate and express 3D space or objects, volumetric visual data capable of expressing geometric information are essential. This information may include geometric shapes and additional information such as color, transparency, and normal vectors. Furthermore, according to the time sequence, if this information is to be expressed in time, information about every moment (individual capture instance) or action is required. Therefore, the temporal expression method may include a method of separately storing information about each moment and recording an object’s movement as a function of time. The former is similar to creating a video by saving still images, and the latter is similar to animating a graphics model. In general, a point cloud is mainly used to express such information.

A point cloud is a set of independent 3D points. Each 3D point contains a 3D position, color, and surface normal. A point cloud can express non-manifold geometry, so it is a more flexible expression method than a polygonal mesh and has the advantage of being processed in real time.

Three-dimensional point cloud data are used in a wide variety of fields. The MPEG PCC standardization activity deals with three categories of point cloud data. The first is a static point cloud, and the second is a dynamic point cloud with temporal information. The third is a dynamically acquired point cloud. MPEG PCC standardization discusses techniques for compressing these point cloud data. These data have (x,y,z) coordinates, and each point has reflectivity and RGB properties.

We deal with human data among dynamic point clouds corresponding to the second data type in MPEG PCC. Just as a temporal sequence of a two-dimensional still image is defined as a video, a dynamic point cloud video is defined as a temporal sequence of a point cloud. We introduce a technique for analyzing human motion and predicting correlation using skeleton information in each frame to compress the point cloud.

### 2.2. Dynamic Point Cloud Capture

**Figure 1:**
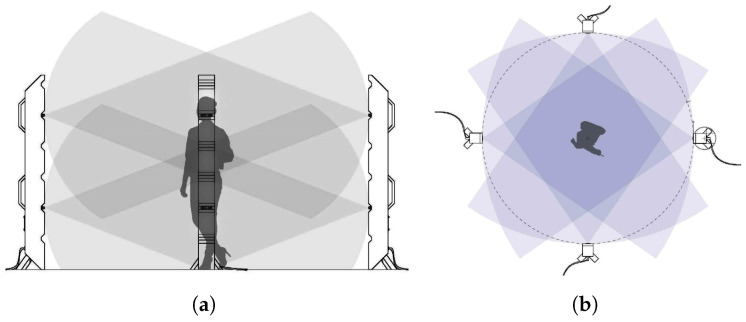
Capturing system of the 3D point cloud sequence: (**a**) side, (**b**) top view.

An RGB-D camera equipped with depth and RGB sensors is used to acquire dynamic point cloud sequences. Since the goal is to generate a volumetric 3D model, eight RGB-D cameras are installed at different viewpoints. Before registering the 3D model, the point cloud is acquired using the depth and RGB images taken through the RGB-D camera. Eight RGB-D cameras are installed on the camera stand. Four sets of stands are placed in the front, back, and side of four directions to capture the object from all directions. Figure 1 shows the camera system to be used.

To calibrate the point cloud captured by multi-view cameras, we minimize errors between feature points of each image. We use the Charuco board to find exact matching points [14] and the gradient descent for transformation parameter optimization [15].

The coordinates for parameter optimization correspond to the internal corner coordinates of the Charuco board. The transformation matrix includes six parameters for rotation and translation about the x, y, and z axes. $X_{ref}$ is the coordinate of the reference camera, and $X_{i}$ is the coordinate of the others. $R_{i\to ref}$ and $t_{i\to ref}$ are the rotation and translation matrices, respectively, and they are initialized at start. The transforming relationship from $X_{i}$ to $X_{i}^{\prime }$ is defined as Equation (1).

(1) $$X_{i}^{\prime }=R_{i\to ref}X_{i}+t_{i\to ref}$$

The loss function used in the parameter optimization is the average value of the squared Euclidean distance between $X_{ref}$ and $X_{i}^{\prime }$. The update process of parameters by the gradient descent is defined as Equation (2) [14,15]. α is a constant value for learning rate and we use 0.1, which was decided in the experiment.

(2) $$P_{n+1}=P_{n}-\alpha \frac{\partial }{\partial P_{n}}(\frac{1}{N}\sum_{j=0}^{N}∥X_{ref(j)}-X_{i}^{\prime }\left(j\right){∥}_{2}^{2})$$

The calibration requires multiple RGB and depth images captured from multi-view RGB-D cameras. The RGB images are used for finding feature points using the Charuco board, and the depth images are used for acquiring the 3D coordinates of the RGB feature points.

### 2.3. 3D Pose Estimation

**Figure 2:**
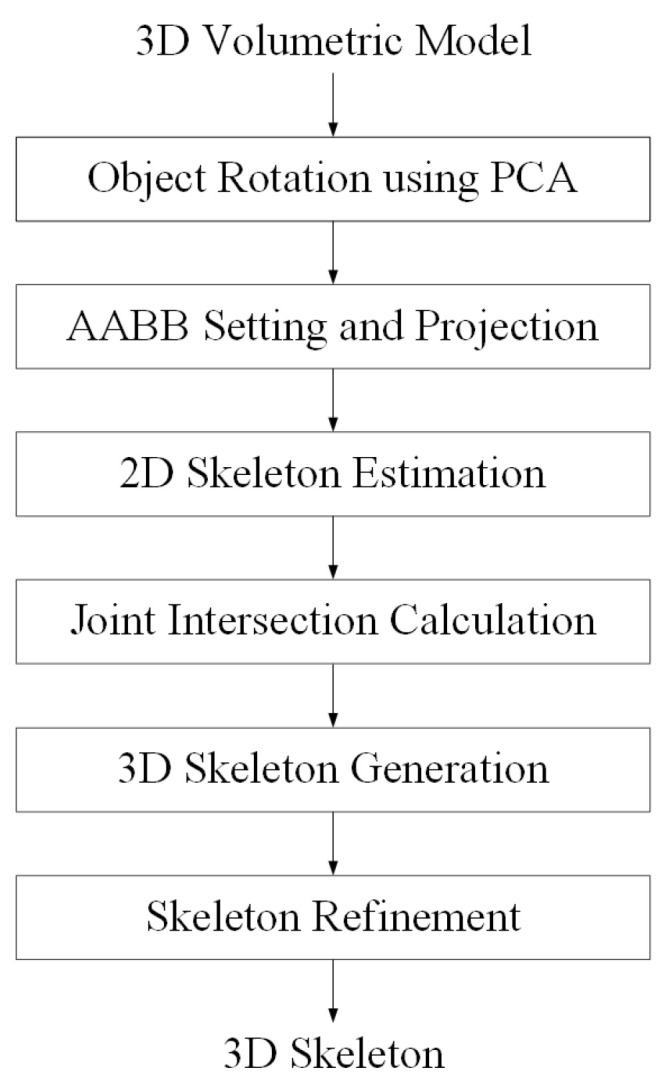
Work flow for 3D skeleton extraction.

When a point cloud is captured through multi-view RGB-D cameras, projection images are generated on four virtual planes for 3D skeleton extraction. Next, the 2D skeleton of the projected image is extracted using the OpenPose library, and the intersection points for the joints in space are calculated for the 3D skeleton operation. We use OpenPose, but it does not matter which method is used to extract the 2D skeleton. Finally, a refinement process for high-precision 3D skeleton extraction is performed. Figure 2 summarizes the algorithm for skeleton extraction.

Since we propose a new algorithm to estimate the motion of the 3D point cloud for compression, we already have a 3D reconstruction result. Therefore, we estimate a 3D human pose in the 3D point cloud domain.

**Figure 3:**
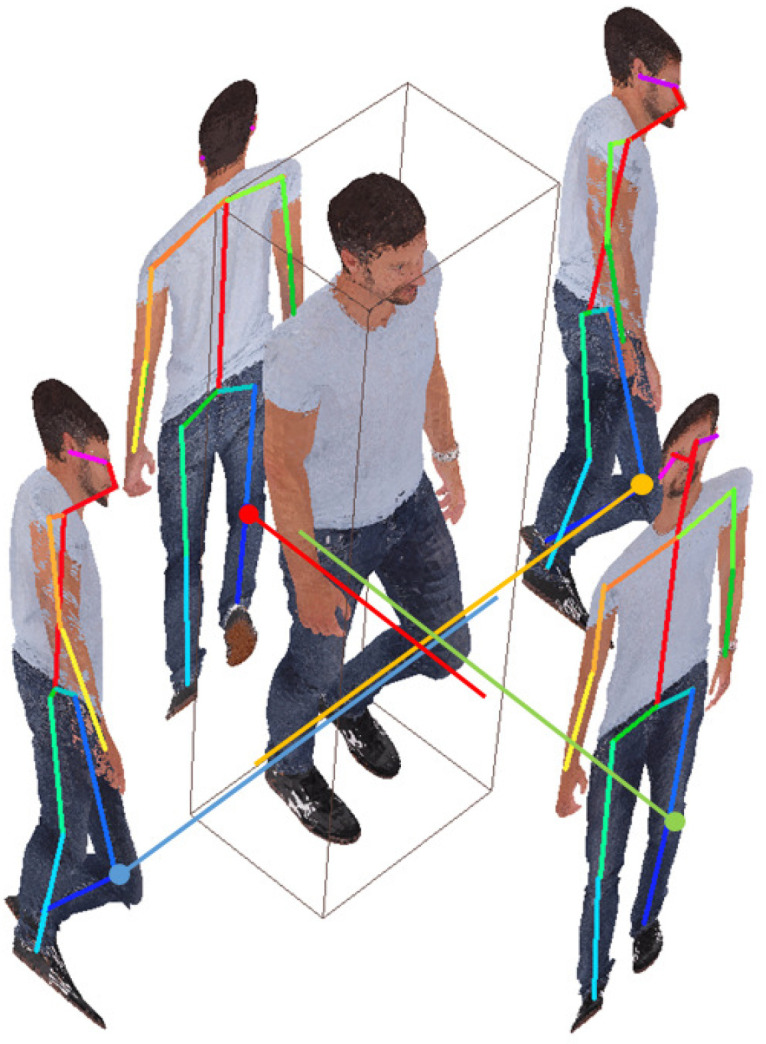
Example of point cloud’s left shoulder 3D joint extraction.

When a 2D skeleton is extracted from the image projected by the point cloud using the OpenPose network, the skeleton extracted from the image projected in the front direction has the highest accuracy. Therefore, the front of the object is found by analyzing the distribution of the point cloud in 3D space, and the front direction of the 3D point cloud is rotated so that it is parallel to the Z-axis direction. Principal component analysis (PCA) is used to find the frontal direction [16]. After finding the front of the object, set the AABB (Axis-aligned Bounding Box) to determine the four projection planes in space [17]. Orthogonal projecting from the 3D to 2D planes uses the MVP (Model View Projection) matrix, which is a 4 × 4 matrix. Using this matrix, the 3D point cloud defined in the 3D world coordinate system is transformed into coordinates on the 2D projection planes which correspond to four sides of AABB. When four projection images are generated, 2D skeletons are extracted using OpenPose [18]. When the skeleton’s 2D coordinate system is restored back to the 3D coordinate system, the joint coordinates extracted on the four projection planes located in space are calculated as shown in Figure 3. If you connect matching coordinates on four planes, you obtain four coordinates intersecting in space. Among these four coordinates, the coordinates having a distance of 3 cm or more from other coordinates are determined as coordinates with errors and removed. In addition to this method, various methods for 3D pose estimation have been introduced, and it does not matter which way is used as long as the accuracy is high. The low accuracy of 3D pose estimation increases the residual point cloud between the two frames’ (key frame, target frame) point clouds, so the compression efficiency can be lowered.

## 3. Temporal Prediction of Dynamic Point Cloud

This section describes our method to estimate the point cloud sequence temporally.

### 3.1. Prediction and Reconstruction

We propose a temporal prediction algorithm for point cloud sequences considering the trade-off relationship between maximizing visual quality and minimizing compressed data to transmit and store a large number of point cloud sequences.

**Figure 4:**
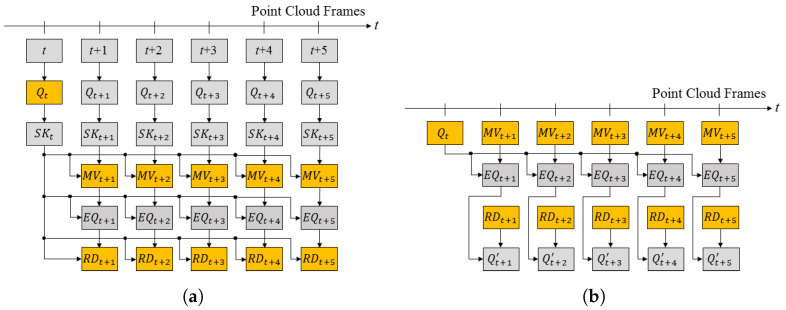
The procedure of coding the point cloud sequence: (**a**) prediction and residual generation; (**b**) reconstruction.

The point cloud sequence (or dynamic point cloud) is a set of point clouds captured at every frame. The 3D skeleton $SK_{t}$ is obtained applying the pose estimation to the point cloud $Q_{t}$ of a frame. The motion vector $MV_{t+1}$ is estimated between the skeletons ($SK_{t}$, $SK_{t+1}$) of the current and next frames. The motion vector is defined as the Euclidian distance between two relative joints with the same number in the hierachical structure of the skeleton. Next, after the deformation ($EQ_{t+1}$) of the 3D point cloud ($Q_{t+1}$ ) of the t+1 frame using motion vector ($MV_{t+1}$), the residual 3D point cloud ($RD_{t+1}$) between the 3D point clouds ($Q_{t}$) of the *t* frame is obtained. The residual PC can be compressed using various methods. Developing an optimal codec for compressing a residual point cloud is beyond the scope of this paper. We focus on the temporal prediction of dynamic 3D point clouds. Finally, $Q_{t}$ (key frame), motion vectors, and the residual 3D point cloud are transmitted. This process is shown in Figure 4a. In Figure 4a, the colored boxes correspond to the transmitted data.

Deforming the key frame $Q_{t}$ using the motion vector $MV_{t+1}$, the deformed model $EQ_{t+1}$ is calculated. Next, the transferred residual data $RD_{t+1}$ are added to $EQ_{t+1}$ and the original 3D point cloud is reconstructed. $EQ_{t+1}$ can be obtained directly from $Q_{t}$ and $MV_{t+1}$ without the need to obtain $SK_{t+1}$, as shown in Figure 4b.

**Figure 5:**
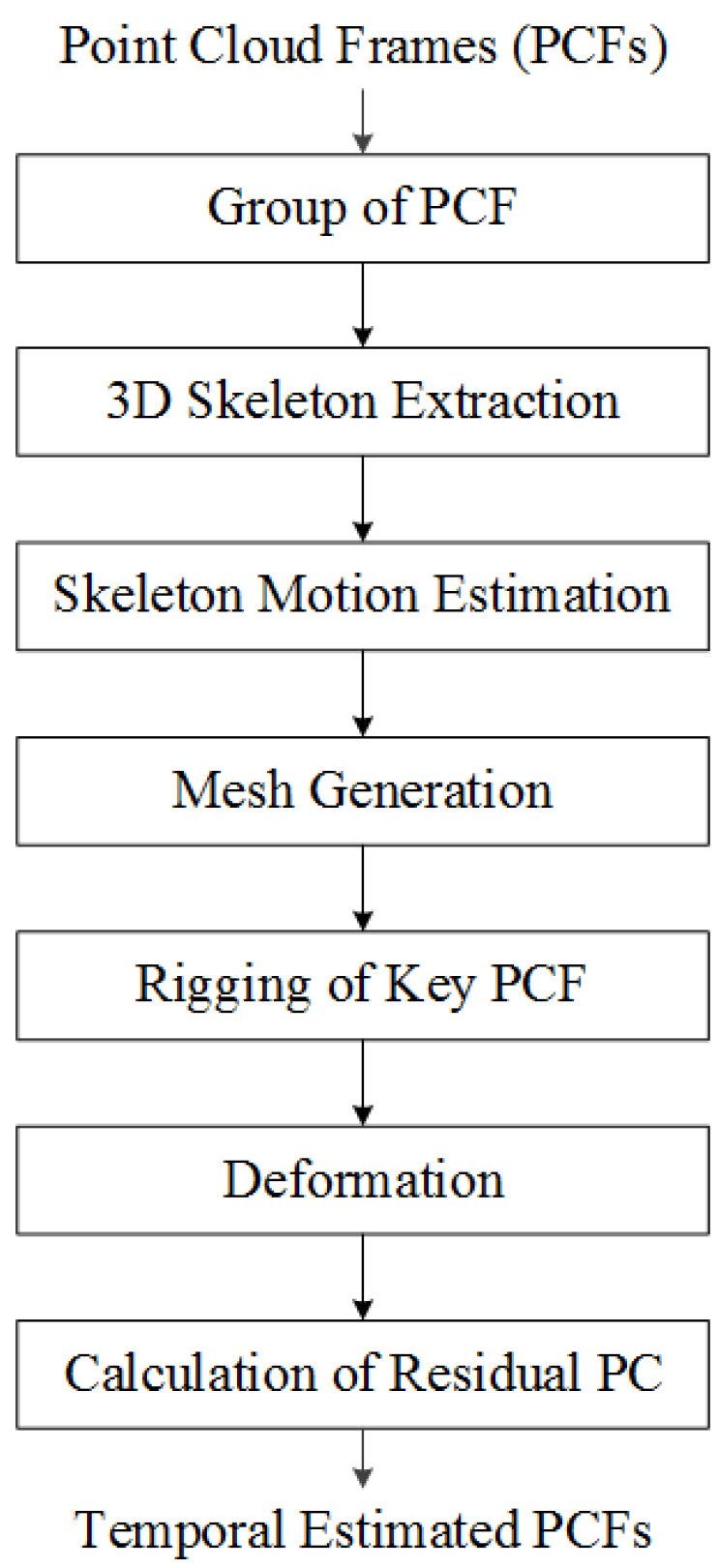
Work flow of proposed point cloud compression algorithm.

Figure 5 summarizes the flow chart for coding the proposed point cloud sequence. When a point cloud sequence is an input, the keyframe interval is determined according to the compression rate. Next, 3D skeletons are extracted from key frames and non-key frames using the proposed algorithm. Next, the keyframe is converted into a mesh for deformation, and the mesh model is deformed using the motion of the skeleton of the non-keyframe. Finally, find the residual between the point cloud composing the deformed mesh and the point cloud of the non-key frame. There are many ways to convert a point cloud into a mesh. Among them, we performed this transformation using the Poisson algorithm [19].

### 3.2. Group of PCF

The first step in Figure 5 is the Group of PCF, where PCF means the point cloud frame in Figure 4. As explained in Section 3.1, PCF prediction is made between point clouds of several frames. This set of frames is defined as a group of PCF, which is a parameter determined by the user.

### 3.3. 3D Skeleton Extraction

**Figure 6:**
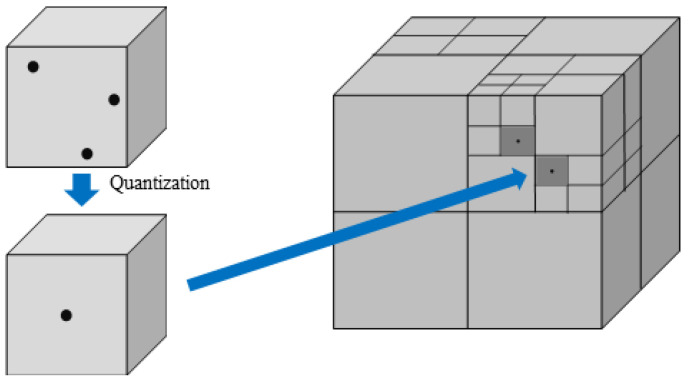
Example of quantization using octree structure.

The second process in Figure 5 is to extract the 3D skeleton. Extracting the 3D skeleton uses the 3D pose estimation algorithm in Section 3.2. After extracting the 3D skeleton, quantize the 3D point cloud to create a deformed mesh based on the extracted 3D skeleton. When calculating the residual of the point cloud, quantization of the coordinate value of the floating point is required because it is necessary to find the location where the coordinates match. An octree, a 3D extended form of a quadtree, has a hierarchical tree structure and a structure in which a parent node is connected to eight child nodes. This paper uses the octree algorithm for quantization [6]. Quantization using the octree structure also affects compression because overlapping points in the 3D model are removed [6]. The minimum unit of voxels is set to 1 mm ${}^{3}$. Figure 6 is an example of a quantization method using an octree structure. As shown in Figure 6, when voxels are divided, all point clouds inside the voxels are converted to the center coordinates of the voxels.

### 3.4. Skeleton Motion Estimation

**Figure 7:**
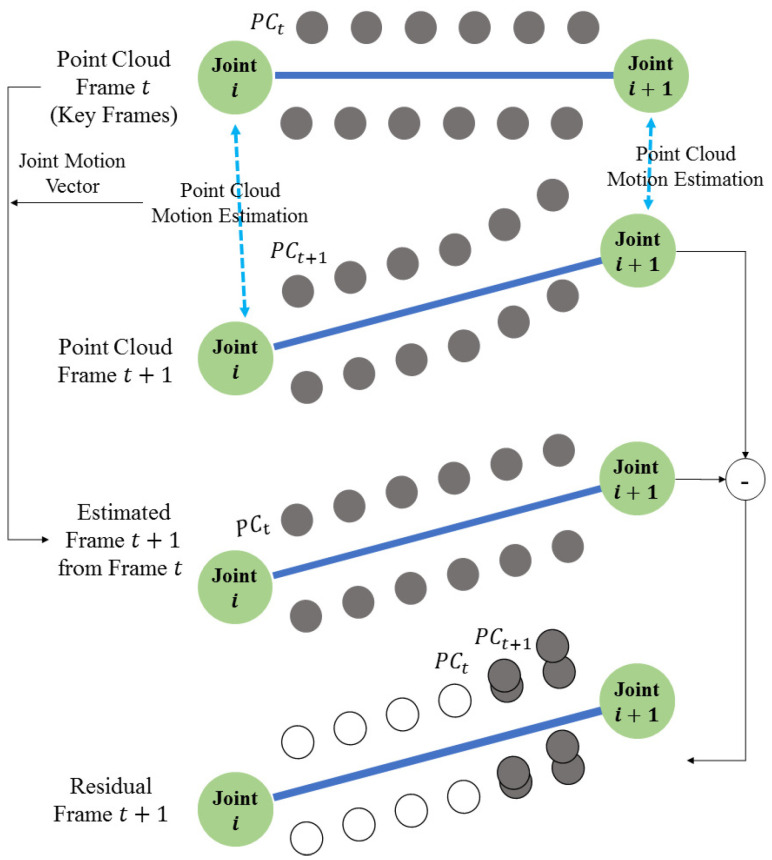
Example of the proposed point cloud compression algorithm.

Figure 7 shows the proposed point cloud estimation algorithm. The point cloud estimation is a technique of predicting a point cloud of the next frame from a point cloud and motion vector of the current frame (or key frame). The point cloud is rigged using the extracted skeleton information for animating it. First, the skeleton (or bones) of the rigged keyframe is animated to the location of the skeleton of the non-key frame and deformed in the form of the point cloud of the non-key frame [20]. The result of animating the point cloud is the deformed point cloud. After deformation, the residual points between the non-key frame and the deformed key frame are obtained. The data transmitted through this process are the initial point cloud of the key frame, the skeleton information of each frame, and data about the point cloud residual of the non-key frame. In Figure 7, the gray-filled circles correspond to the available point.

### 3.5. Deformation of 3D Point Cloud

Before estimating the motion of the point cloud sequence, the threshold for the amount of the residual point cloud is required to decide the key frame. A keyframe is determined using information about the number of residual point clouds. The number of key frames can be changed according to the compression ratio. A keyframe is determined as a frame in which point clouds with other frames may overlap a lot. The other frames, except the key frames, transfer the motion vector of the 3D skeleton and the residual point cloud. If the threshold is larger, the interval of key frames is larger. If the key frame increases in a sequence, the compression ratio decreases, and the visual quality increases.

**Figure 8:**
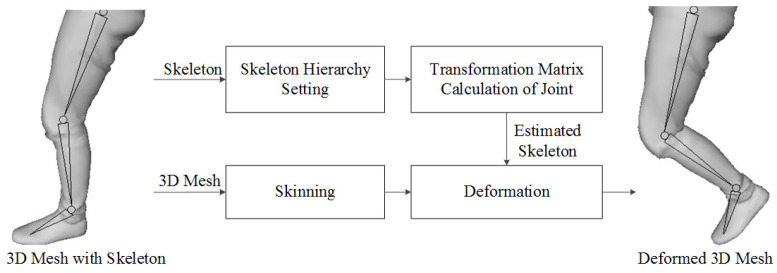
Mesh deformation workflow.

Next, we explain the deformation step for motion compensation. To compensate for the 3D PC, after deforming the 3D key PCF using the estimated motion vector by the 3D pose estimation, the overlapped PC between the key and target PCFs is removed using the motion-compensated 3D key PCF. Next, the 3D PC is converted to the mesh model for deformation, where we use Poisson surface reconstruction [21]. Figure 8 is a flow for the mesh deformation to convert mesh using the 3D mesh and skeleton. After generating the 3D mesh, skinning, which attaches the skin to bones using the estimated skeleton, is conducted. The transformation matrix is also required to deform the 3D key PCF, which calculates the 3D distance and direction between the joints of the key PCF and target PCF. The matrix and estimating method are the same types as Equation (1). The key PCF is deformed to the target PCF using the transformation matrices of the joints and the skinned 3D model.

**Figure 9:**
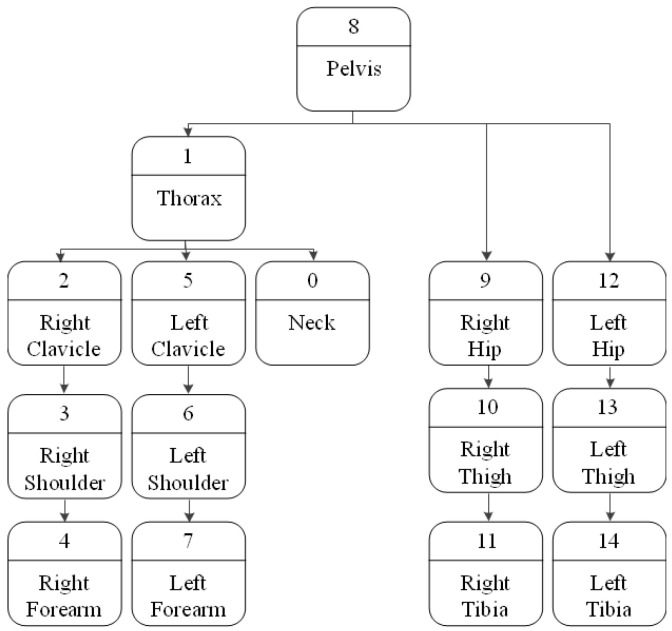
Skeleton hierarchy.

When a person moves, the movement of one part may or may not affect the movement of another part. For example, if you move your thigh up, your calves will be affected by the movement of your thigh and move up together. However, just because the calf moves does not necessarily mean that the thigh moves with it. A hierarchical structure is set in the 3D model to reflect this influence so that the lower nodes work together with the movement of the upper nodes. In the skeleton hierarchy, the pelvis corresponding to the core is the highest node, and the closer it is to the pelvis, the higher the node is. Figure 9 shows the skeleton hierarchy used [21].

**Figure 10:**
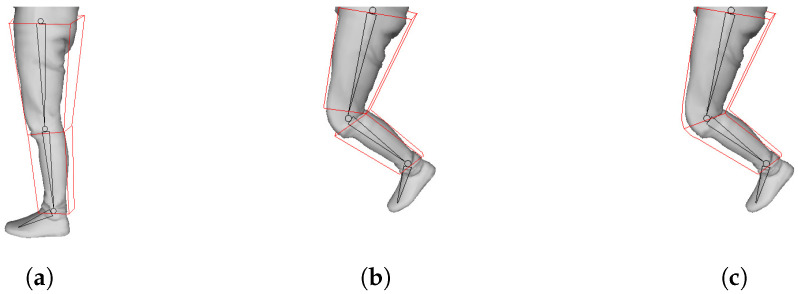
Skinning example: (**a**) keyframe mesh and skeleton that has been skinned; (**b**) keyframe mesh and skeleton before and (**c**) after skinning weight is applied.

After setting the hierarchy, skinning is performed from the skeleton of the lower hierarchy. We use the bisector of the two bones. First, a mesh is assigned to the bones located on the left and right with respect to the plane passing through the bisector. However, if the mesh is divided simply like this, the mesh may be separated when it is moved. Therefore, skinning weight is used to express natural movement [22,23]. Since the degree of skin stretching can vary depending on the distance away from the joint, this part is expressed using skinning weights. Figure 10 is an example of the movement of the mesh before and after applying the skinning weight. Figure 10a is a key frame mesh and skeleton to be transformed, Figure 10b is a non-key frame mesh and a keyframe mesh transformed without weight, Figure 10c is a non-key frame mesh, and a key frame mesh with weights applied. This is an example of deformation when applied. From this figure, you can see that the joint is deformed unnaturally if no weight is applied. The weight has a value between 0 and 1, and the closer it is to the center of the bone, the closer it gets to 1, and the closer it gets to the joint, the closer it gets to 0 [23].

**Figure 11:**
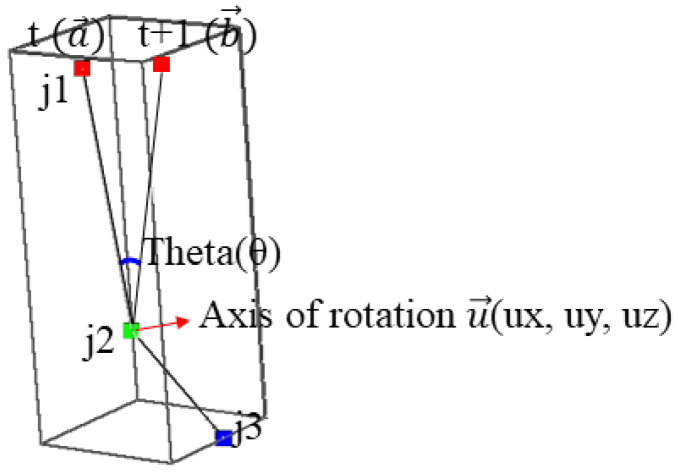
Examples of Skeleton Coordinate Transformation.

Next, we explain calculation of the transformation matrix (R|T) for the joint. The movement of a bone from the frame t to the frame *t* + 1 is shown in Figure 11. j1, j2, and j2 represent joints. $t(\vec{a})$ is the direction vector from j2 to j1 in the frame t and $t+1(\vec{b})$ is the direction vector from j2 to j1 in the frame *t*+1. θ is an angle between $t(\vec{a})$ and $t+1(\vec{b})$, and $\vec{u}$ is an axis of rotation.

The transformation matrix (*T*) is derived from the joint movement defined as Equation (3).

(3) $$T=j2_{t+1}-j2_{t}$$

The rotation matrix (*R*) can be estimated from the angle and axis of rotation. In Figure 11, the 3D coordinate of j2 is fixed in t and t+1, but it can be changed. After assuming that $j2_{t+1}$ and $j2_{t}$ are located in the same position, the rotation matrix is calculated. The axis of rotation ($\vec{u}$) is calculated as the cross product of $t(\vec{a})$ and $t+1(\vec{b})$ as shown in Equation (4).

(4) $$\vec{u}=t+1\left(\vec{b}\right)\times t\left(\vec{a}\right)$$

The angle of rotation is calculated by the arccosine of the inner product of $t(\vec{a})$ and $t+1(\vec{b})$ as Equation (5).

(5) $$\theta =a\cos(t+1\left(\vec{b}\right)•t\left(\vec{a}\right))$$

The rotation matrix is defined as Equation (1) using the axis and angle of rotation.

(6) $$R=\left[\begin{array}{ccc} \cos\theta +u_{x}^{2}\left(1-\cos\theta \right) & u_{x}u_{y}\left(1-\cos\theta \right)-u_{z}\sin\theta & u_{x}u_{z}\left(1-\cos\theta \right)+u_{y}\sin\theta \\ u_{y}u_{x}\left(1-\cos\theta \right)+u_{z}\sin\theta & \cos\theta +u_{y}^{2}\left(1-\cos\theta \right) & u_{y}u_{z}\left(1-\cos\theta \right)-u_{x}\sin\theta \\ u_{x}u_{z}\left(1-\cos\theta \right)-u_{y}\sin\theta & u_{y}u_{z}\left(1-\cos\theta \right)+u_{x}\sin\theta & \cos\theta +u_{z}^{2}\left(1-\cos\theta \right) \end{array}\right]$$

The mesh (or point cloud) is deformed with the skinning weight and the transformation matrix. The mesh is deformed with Equation (7), where *W* is the skinning weight, and *X* and $X^{\prime }$ are the coordinates after and before deformation, respectively.

(7) $$X^{\prime }=W\left(R\left(X-j2_{t}\right)+j2_{t+1}\right)$$

### 3.6. Residual Point Cloud

**Figure 12:**
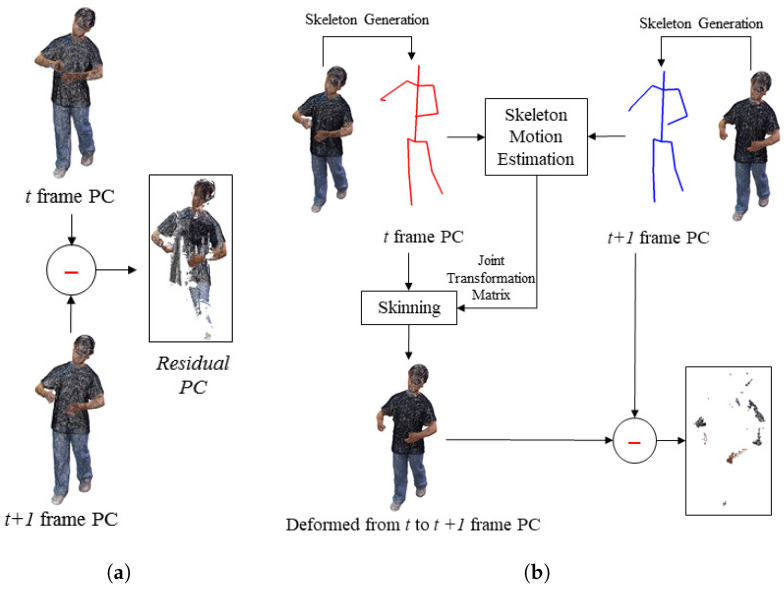
3D point cloud compression process using skeleton estimation: (**a**) frame-by-frame residual calculation method; (**b**) proposed method.

This section describes the process of finding the residuals. Since we are dealing with a point cloud, we return the vertices of the mesh to the point cloud. The vertices of the mesh model are treated as the same information as the 3D PC. The mesh model can be viewed as the same as the 3D PC model. Using the method described in the previous section, the deformed target PCF is calculated using the key PCF and the target PCF, and the residual between the original target PCF and the deformed target PCF is calculated. Figure 12 conceptually shows how to calculate the residual of the point cloud before and after pose estimation and deformation. In the case of calculation, as shown in Figure 12a, most of the upper body remains residual. However, as shown in Figure 12b, the number of residual point clouds can be reduced by more than three times by using the proposed method. In addition, lossless compression, such as binary encoding, is applied to the residual point cloud. If various compression codecs, including methods such as MPEG PCC, are applied to the residual, a higher compression rate can be obtained. However, since we conducted a study to reduce the number of point clouds themselves, this paper needs to discuss such compression techniques in detail. Also, how such a compression technique can affect our proposed method is not covered, as it is beyond the focus of this paper.

## 4. Experimental Result

**Figure 13:**
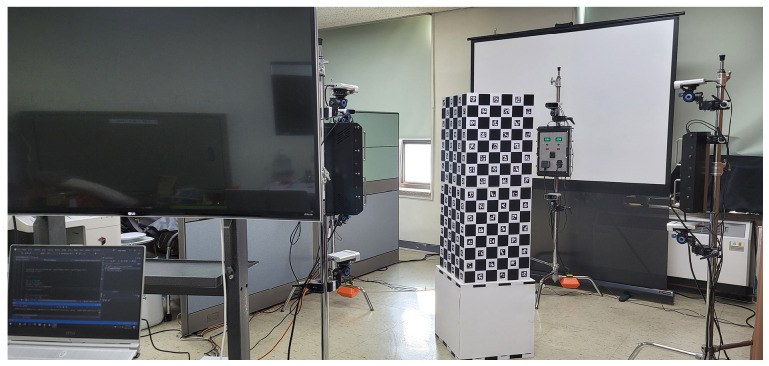
Camera system environment used.

In this section, the experimental environment and experimental results are explained. Eight Microsoft Kinect Azure cameras were used. The camera arrangement follows the shooting system, as shown in Figure 13. Four units were installed at 0.7 m from the ground, and the remaining four units were placed at 1.5 m from the floor to photograph the top of the object. A threshold value was set for the depth value to obtain a point cloud for objects within 0.1 m to 1.5 m. Figure 13 is a photograph of the configured camera system.

**Figure 14:**
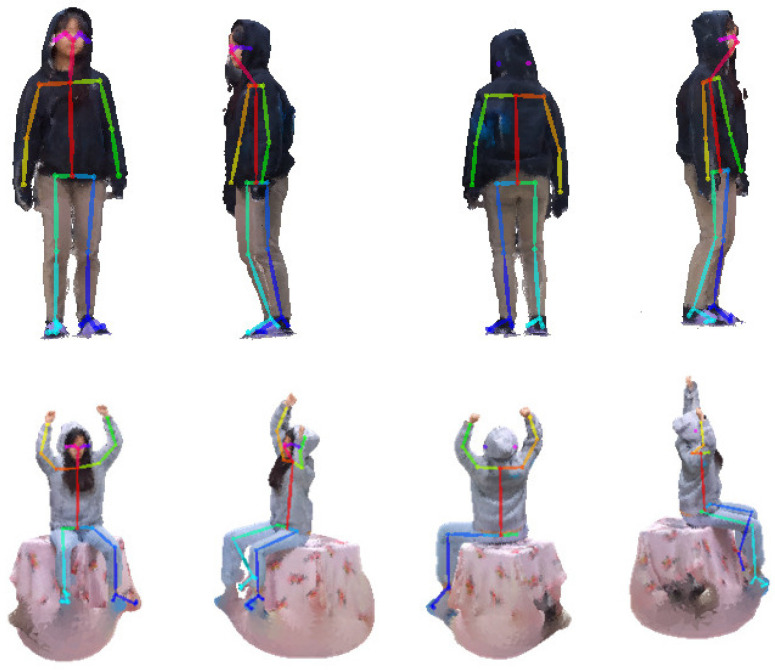
Extraction of the 2D skeleton of the projected image: (**a**) front, (**b**) right, (**c**) rear, and (**d**) left.

Figure 14 shows projection images of the two 3D point cloud sequences used in the experiment and the result of extracting the 2D skeleton from the projection images. The upper 3D point cloud sequence has a walking motion, and the lower 3D point cloud sequence is stationary and sitting. The upper sequence features most of the body visible from all sides, while the lower sequence features an occlusion where parts of the legs are not visible from the back. As shown in Figure 14, the skeleton extraction is more accurate on the front than on the other side.

**Figure 15:**
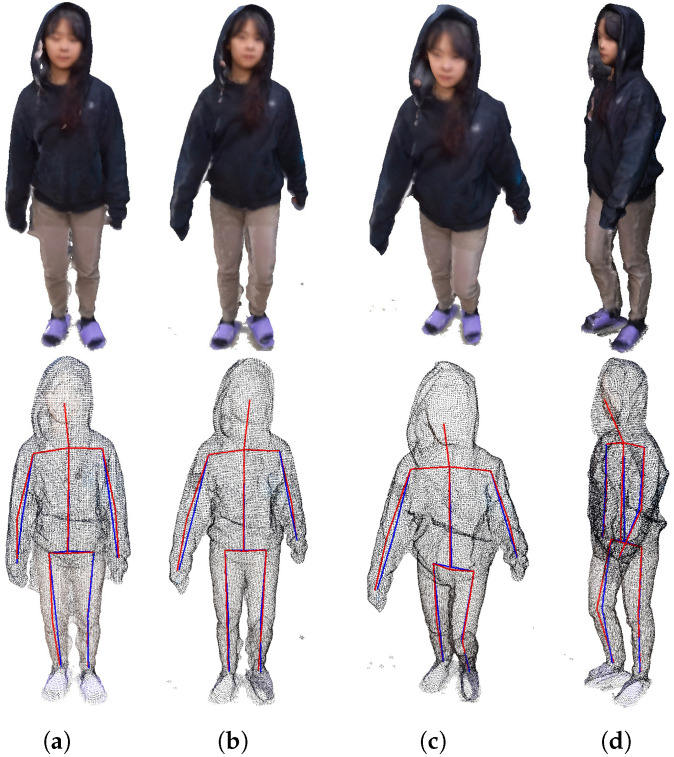
For the 4 frames acquired directly, (**a**) the first frame, (**b**) the second frame, (**c**) the third frame, (**d**) the mesh of the fourth frame and the skeleton before and after post-processing.

Figure 15 shows four frames from the first 3D point cloud sequence in Figure 14. In Figure 15, the first row represents the texture of the 3D model, and the second row represents the point cloud. The result of estimating the 3D skeleton from the four extracted frames is shown. If you check the estimated 3D skeleton, you can visually confirm that all joints and bones are accurately located inside the point cloud distribution of the model.

**Figure 16:**
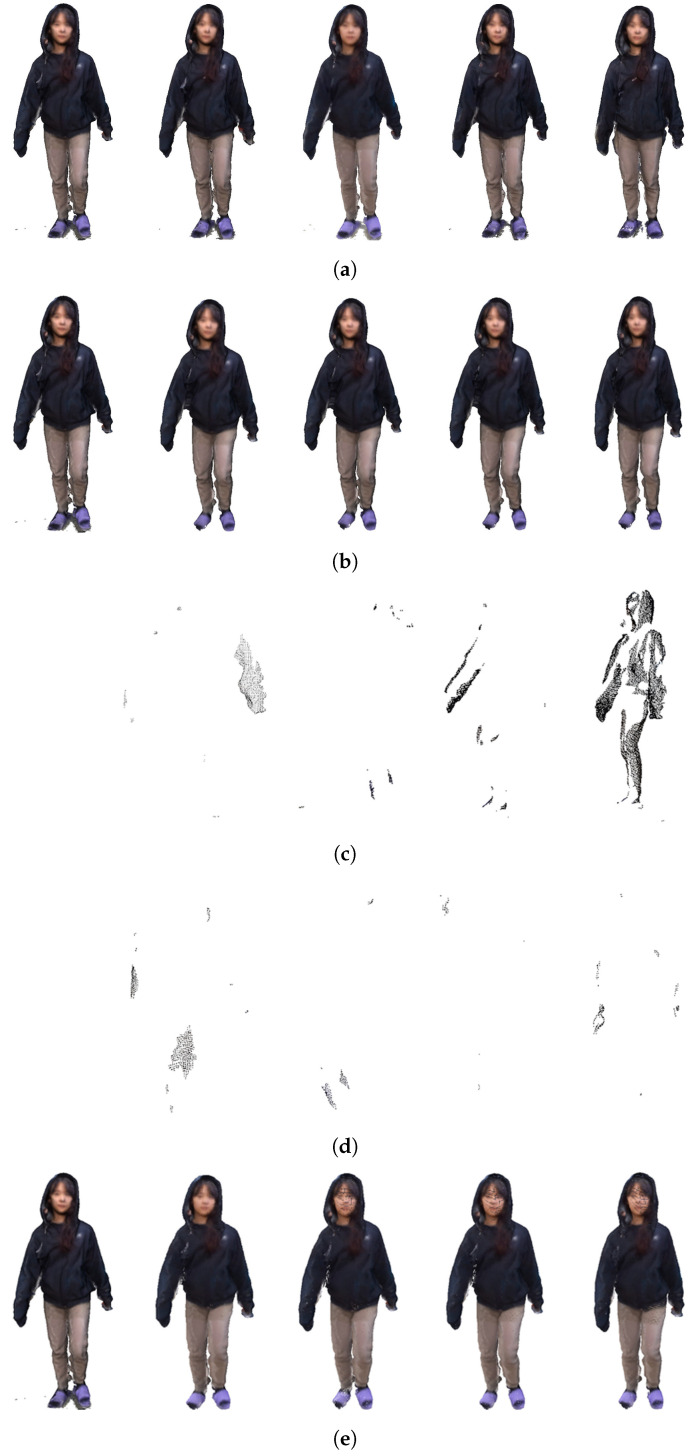
Compression and reconstruction result: (**a**) original pointcloud, (**b**) mesh deformation result, (**c**) residual result to which estimation was not applied, (**d**) residual result to which estimation was applied, (**e**) restoration result.

Compression was performed on five frames of directly acquired point cloud data. The first frame was keyframed, and the rest were compressed. Figure 16 is the compression result of five frames. Figure 16a is the mesh before compression, Figure 16b is the deformed mesh after skinning, Figure 16c is the point cloud residual obtained without motion compensation, Figure 16d is the point cloud residual after motion compensation, and Figure 16e is the restored point cloud result. Figure 16 shows that the compression effect improves as the sequence increases.

Table 1 shows the number of vertices and data size between the original and compressed data after applying octree quantization, the proposed compression algorithm, and the binary encoding of each frame. This table shows the results in three ways. The first is for developing the original frames, and the second is the compression result using the residual point clouds by the prediction between the key and non-key frames. After prediction and deformation, the final compression result is calculated by the residual point clouds. To the results of this table, the compression rate before and after applying motion compensation increased more than three times. The point cloud quantization was performed with the small size of a voxel. The criterion of the voxel size is that the result of quantization is visually unaffected. The numerical effect of quantization is included in the compression result.

For performance evaluation, a tool called cloud compare was used to measure the error’s mean distance and standard deviation, which have the unit of meter, between the original point cloud and the restored point cloud after compression. Table 2 is the Mean Distance and Standard Deviation measurement results using Cloud Compare. The mean distance of all frames is within ±0.1, and the standard deviation is less than 0.05, which does not differ significantly from the original data [24].

## 5. Conclusions

We propose a system for acquiring a photo-realistic-based point cloud sequence at 30 fps through an RGB-D camera system to extracting a 3D skeleton and compressing it. To extract the skeleton, create a projection plane for the four sides of the object and extract the 2D skeleton using the Openpose library, a deep learning model. Then, post-processing is performed for high-precision skeleton extraction. The 3D skeleton for the entire point cloud of the extracted sequence is used for compression. Compression is carried out in the form of moving the rigged keyframe to the skeleton movement of the non-keyframe, predicting the movement, removing overlapping points, and transmitting only the residual.

Using eight RGB-D cameras, it was possible to acquire point cloud data based on real-life images in all directions. In addition, it was possible to create an integrated point cloud with an error of less than 5 mm through the camera calibration process by applying the optimization algorithm. In addition, it was possible to extract a high-precision 3D skeleton without additional motion capture equipment using the generated point cloud sequence. A stable skeleton with half the standard deviation was extracted through a post-processing algorithm to compensate for the instability of the deep learning network. In addition, using this skeleton, the number of residual point clouds can be reduced by about 12 times by predicting motion between point clouds and removing temporally overlapping points. In addition, motion compensation increased the compression rate three times, and the compression effect improved as the sequence increased. In addition, the restored data after compression through Cloud Compare were similar to the original, with a mean distance of ±0.1 and a standard deviation of less than 0.05.

## Figures and Tables

**Table 1:** Number of vertices and data amount before and after compression for each frame.

Item	Frame	Key Frame	Non-Key Frame	Ratio
Original	Number of Point Cloud	348,597	341,334	100.00%
	Data size (KB)	17,699	16,427	100.00%
Residual	Number of Point Cloud	348,597	26,473	7.76%
	Data size (KB)	17,699	1061	6.46%
Residual with Deformation	Number of Point Cloud	348,597	2190	0.64%
	Data size (KB)	6923	35	0.01%

**Table 2:** Mean Distance and Standard Deviation measurement results using Cloud compare.

Frame	t + 1	t + 2	t + 3	t + 4
Mean Distance (m)	0.004571	0.006422	0.009824	0.014579
Standard Deviation (m)	0.002758	0.00506	0.007838	0.009799

## Data Availability

Not applicable.
